# A Coherence Preservation Control Strategy in Cavity QED Based on Classical Quantum Feedback

**DOI:** 10.1155/2013/340917

**Published:** 2013-05-28

**Authors:** Ming Li, Wei Chen, Junli Gao

**Affiliations:** School of Automation, Guangdong University of Technology, No. 100 Waihuan Xi Road, Guangzhou Higher Education Mega Center, Pan Yu District, Guangzhou, Guangdong 510006, China

## Abstract

For eliminating the unexpected decoherence effect in cavity quantum electrodynamics (cavity QED), the transfer function of Rabi oscillation is derived theoretically using optical Bloch equations. In particular, the decoherence in cavity QED from the atomic spontaneous emission is especially considered. A feedback control strategy is proposed to preserve the coherence through Rabi oscillation stabilization. In the scheme, a classical quantum feedback channel for the quantum information acquisition is constructed via the quantum tomography technology, and a compensation system based on the root locus theory is put forward to suppress the atomic spontaneous emission and the associated decoherence. The simulation results have proved its effectiveness and superiority for the coherence preservation.

## 1. Introduction

The enormous potential of quantum information has caused the widespread concern in the scientific community and has become an important research focus. Among the implementation of hardware design for quantum computing such as cavity QED, ion trap, nuclear magnetic resonance, quantum dots, and superconducting systems [1], cavity QED is one of the most promising schemes because the basic interaction within cavity QED is the vacuum Rabi oscillation and the strong coupling of cavity field and atom allows atom-photon system to maintain good quantum coherence within the time scale of the kinetic characteristics. Therefore, a variety of entangled state preparation methods have been proposed based on cavity QED. Accordingly, the advantages of cavity QED have made it possible to construct decisive multiparticle entanglement in experiment using it [2, 3].

However, all the advantages in cavity QED depend on the coherence of the system. The loss of coherence in quantum mechanical superposition states limits the time for which quantum information remains useful. Similarly, it limits the distance over which quantum information can be transmitted [4]. Hence, decoherence is the major obstacle that hinders the processing of quantum information in various physical implementations [5]. The preservation of quantum coherence is of fundamental importance in the hardware implementation of quantum information. In cavity QED, the foundation of quantum information processing is the Rabi oscillation, an undamped oscillation process, which can be destroyed by the spontaneous emission of the atom. Thus, aiming at eliminating the decoherence effects in cavity QED, a classical feedback control strategy is presented based on the transfer function of the Rabi oscillation. A feedback channel is constructed through the quantum tomography technique for the quantum information acquisition to obtain the Rabi oscillation stabilization control law based on the root locus theory, which can be used to suppress the atomic spontaneous emission and the associated decoherence effect. Finally a physical implementation scheme of the strategy is given and the simulation results show that it can prevent the Rabi oscillation from vanishing and thus provide a stable environment for quantum computing.

## 2. Methods

### 2.1. The System Model

**Figure 1:**
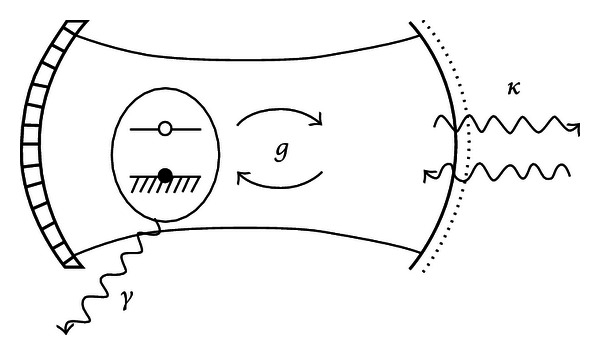
Schematic presentation of cavity QED [6].

The cavity QED investigates the interaction of single atoms with single electromagnetic field modes, defined, for example, by a pair of mirrors illustrated in Figure 1 [6], which is a schematic representation of a cavity QED system consisting of an atom with two energy levels interacting with a single photon mode trapped by mirrors to form a cavity. A photon in the cavity, bouncing back and forth between the mirrors, can be absorbed by the atom; conversely, if the atom is excited, it can decay by emitting a photon into the cavity. The rate of this atom-light interaction (*g*) is proportional both to the dipole moment of the atom and to the electric field of the photon at the atom's location.

The strong-coupling regime is reached when the interaction rate of the atom and a single photon (*g*) is larger than the dissipation arising from the loss of photons (at rate *κ*) or from emission from the atom into other modes at rate *γ*, that is, *g* ≫ *κ*, *γ*. The excited atoms will periodically release and absorb photons with a certain frequency, a phenomenon known as vacuum Rabi oscillations [7]. The presence of the cavity has made the spontaneous emission from the atom, usually an irreversible process, into a coherent and reversible oscillation, which means that quantum information can be exchanged back and forth between the atom and the photon many times before it is lost forever [6].

Undesired processes can take place in any real system and the challenge for realizing strong-coupling cavity QED and the generation of entanglement is to maximize the vacuum Rabi frequency while simultaneously to minimize the decoherence effects described by the decay (*κ*, *γ*). In this paper, the optical Bloch equations describing the mechanism of cavity QED are investigated to obtain the transfer function of the Rabi oscillation with the spontaneous emission [8]. A compensation strategy is designed to suppress the spontaneous emission of atoms, which has a negative effect on the coherence of the system. It should be noted that all of the following discussion is based on zero detuning as a precondition.

Now we start from the optical Bloch equation of the cavity QED. The time-dependent mechanics of cavity QED is based on the time-dependent Schrödinger equation [9]:

(1) $$\begin{array}{c} \hat{H}\psi \left(r,t\right)=i\hbar \frac{\text{d}\psi \left(r,t\right)}{\text{d}t}. \end{array}$$

We first investigate an isolated 2-level system |*ψ*_1_〉,  |*ψ*_2_〉 with energy eigenvalues *E*_1_,  *E*_2_. The energy difference is related to the transition frequency 2*πω*_0_, that is, *ℏω*_0_ = *E*_2_ − *E*_1_. Then we move forward to consider the effect on the atom of an incident light beam. Their interaction with the atom causes an additional electromagnetic energy *H*_*I*_ to the Hamiltonian of the system *H*_0_. The total Hamiltonian is then *H* = *H*_0_ + *H*_*I*_, which is explicitly dependent on time. In this case, specifying the vector of the light field **E**_0_ pointing to the *x*-direction, if the frequency *ω* of the light is close to *ω*_0_, only the two selected atomic states are involved in the radiative process. The solution to the Schrödinger equation (1) must be a linear superposition

(2) $$\begin{array}{c} \psi \left(r,t\right)=C_{1}\psi_{1}\left(r,t\right)+C_{2}\psi_{2}\left(r,t\right) \end{array}$$

with ∫d*V*|*ψ*(**r**,*t*)|^2^ = |*C*_1_|^2^ + |*C*_2_|^2^ = 1.

Assume that the vector of the light field is **E**_0_, and the total atomic electric-dipole moment is **J** = −*e*∑**r**_*k*_ = −*e* **D**. The main contribution to the interaction Hamiltonian arises from the potential energy of this electric dipole in the electric field of the light beam. We can write

(3) $$\begin{array}{c} H_{I}=eD\cdot E_{0}\cos\omega t. \end{array}$$

Inserting (2) into (1), one obtains the equations for the coefficients *C*_1_(*t*) and *C*_2_(*t*):

(4) C1M11+C2e−iω0tM12=idC1dt,C1eiω0tM21+C2M22=idC2dt,

where *M*_*km*_ are the transition matrix elements: *ℏM*_*km*_ = ∫*ψ*_k_*H_*i*_ *ψ*_*m*_d*V* = 〈*ψ*_*k*_|*H*|*ψ*_*m*_〉. From symmetry one sees *M*_11_ = *M*_22_ = 0 and *M*_12_ = *M*_21_* = (1/*ℏ*)**e** · **E**_0_ *X*_12_cos⁡⁡(*ωt*) where $X_{12}=\int \psi_{1}^{*}X\psi_{2}\text{d}V=\langle \psi_{1}|\hat{H}|\psi_{2}\rangle$ is the dipole matrix element. Now, we define the Rabi frequency *Ω* as

(5) $$\begin{array}{c} \Omega =\frac{1}{\hbar }e\cdot E_{0}X_{12}. \end{array}$$

Using these matrix elements, (4) can be now written as

(6) $$\begin{array}{c} \Omega \cos\omega te^{-i\omega_{0}t}C_{2}=i\frac{\text{d}C_{1}}{\text{d}t},\\ \Omega^{*}\cos\omega te^{i\omega_{0}t}C_{1}=i\frac{\text{d}C_{2}}{\text{d}t}. \end{array}$$

For *ω*_0_ + *ω* ≫ *ω*_0_ − *ω*, we can neglect the fast oscillation terms (*ω* + *ω*_0_)*t*. The evolution will be governed by the slow oscillating terms. This approximation is called the Rotating Wave Approximation (RWA).

Using cos⁡⁡*ωt* = (*e* ^*i* *ωt*^ + *e* ^−*i* *ωt*^)/2, (6) can be rewritten as

(7) $$\begin{array}{c} \Omega \frac{1}{2}e^{-i(\omega -\omega_{0})t}C_{2}=i\frac{\text{d}C_{1}}{\text{d}t},\\ \Omega^{*}e^{i(\omega -\omega_{0})t}C_{1}=i\frac{\text{d}C_{2}}{\text{d}t}. \end{array}$$

For zero detuning, *ω* = *ω*_0_, one finds the well-known Rabi oscillations between the ground and excited states of the driven two-level system.

From the coefficients *C*_1_(*t*) and *C*_2_(*t*), we can form equations for the density matrix of the atom. Noticing the four elements of the atomic density matrix element *ρ*_*ij*_ defined by *ρ*_11_ = |*C*_1_|^2^, *ρ*_12_ = *C*_1_ *C*_2_*, *ρ*_21_ = *C*_1_*C_2_ = *ρ*_12_*, *ρ*_22_ = |*C*_2_|^2^ with the real diagonal elements satisfying *ρ*_11_ + *ρ*_22_ = 1 and the off diagonal elements satisfying *ρ*_12_ = *ρ*_21_*, one can apply the RWA to find the equations of motion for the density matrix as follows:

(8) $$\begin{array}{c} \frac{\text{d}\rho_{22}}{\text{d}t}=-\frac{\text{d}\rho_{11}}{\text{d}t}=-\frac{1}{2}i\Omega^{*}e^{i(\omega_{0}-\omega )t}\rho_{12}+\frac{1}{2}iAe^{-i(\omega_{0}-\omega )t}\rho_{21},\\ \frac{\text{d}\rho_{12}}{\text{d}t}=-\frac{\text{d}\rho_{21}^{*}}{\text{d}t}=\frac{1}{2}i\Omega e^{-i(\omega_{0}-\omega )t}\left(\rho_{11}-\rho_{22}\right). \end{array}$$

The previous discussions are concerned with the situation when there is no damping due to spontaneous emission. Now we consider the spontaneous emission; the rectified equations are as follows:

(9) dρ22dt=−dρ11dt=−12iΩ∗ei(ω0−ω)tρ12+12iΩe−i(ω0−ω)tρ21−2γρ22,dρ12dt=−dρ21∗dt=12iΩe−i(ω0−ω)t(ρ11−ρ22)−γρ12,

where *γ* is the atomic spontaneous emission damping ratio.

For the special initial conditions *ρ* = [1,0,0,0]′ in the case of resonant light *ω* = *ω*_0_, the optical Bloch equation degenerates into constant coefficient differential equations [9]:

(10) dρdt=[02γ12iΩ∗−12iΩ0−2γ−12iΩ∗12iΩ−12iΩ12iΩ−γ012iΩ∗−12iΩ∗0−γ]ρρ(0)=[1000]′.

Noticing that *ρ*_11_ = *ρ*_11_*, *ρ*_22_ = *ρ*_22_*, we can give a general solution of *ρ*_22_:

(11) $$\begin{array}{c} \rho_{22}=\frac{{\left|\Omega \right|}^{2}}{4\gamma^{2}+2{\left|\Omega \right|}^{2}}\left[1-\left(\cos\mu t+\frac{3\gamma }{2\mu }\sin\mu t\right)e^{-3\gamma t/2}\right] \end{array}$$

(12) $$\begin{array}{c} \mu =\sqrt{{\left|\Omega \right|}^{2}-\frac{1}{4}\gamma^{2}}. \end{array}$$

### 2.2. The Rabi Oscillation Stabilization

When external coherent laser field was applied, the vacuum-field-induced coherence effects will be replaced by the microwave-field-induced coherence effects. The decoherence effect caused by spontaneous emission in the system can be suppressed by the introduction of the control of the laser field. Furthermore, the method for implementing the decoherence suppression is to change the Rabi oscillation frequency. According to the previous strategy, the transfer function of the system is constructed and then the decoherence suppression is realized through the compensation of the transfer function.

We can infer from (11) that the underdamped Rabi oscillation is a typical second-order system and the open-loop transfer function is (0 < *ξ* < 1):

(13) $$\begin{array}{c} G\left(s\right)=\frac{K_{0}\omega_{n}^{2}}{s^{2}+2\xi \omega_{n}s+\omega_{n}^{2}}. \end{array}$$

Hence, the unit step response of the open-loop system described by (10) is as follows:

(14) $$\begin{array}{c} y\left(t\right)=K_{0}\left[1-\left(\cos\omega_{d}t+\frac{\xi }{\sqrt{1-\xi^{2}}}\sin\omega_{d}t\right)e^{-\xi \omega_{n}t}\right],\\ \omega_{d}=\sqrt{1-\xi^{2}}\omega_{n}. \end{array}$$

Comparing formula (11) and (14), the parameters in the transfer function of damping Rabi oscillation can be obtained as follows:

(15) $$\begin{array}{c} K_{0}=\frac{{\left|\Omega \right|}^{2}}{4\gamma^{2}+2{\left|\Omega \right|}^{2}}, \end{array}$$

(16) $$\begin{array}{c} \xi =\frac{3\gamma }{\sqrt{4\mu^{2}+9\gamma^{2}}},\\ \omega_{n}=\frac{1}{2}\sqrt{4\mu^{2}+9\gamma^{2}}. \end{array}$$

**Figure 2:**
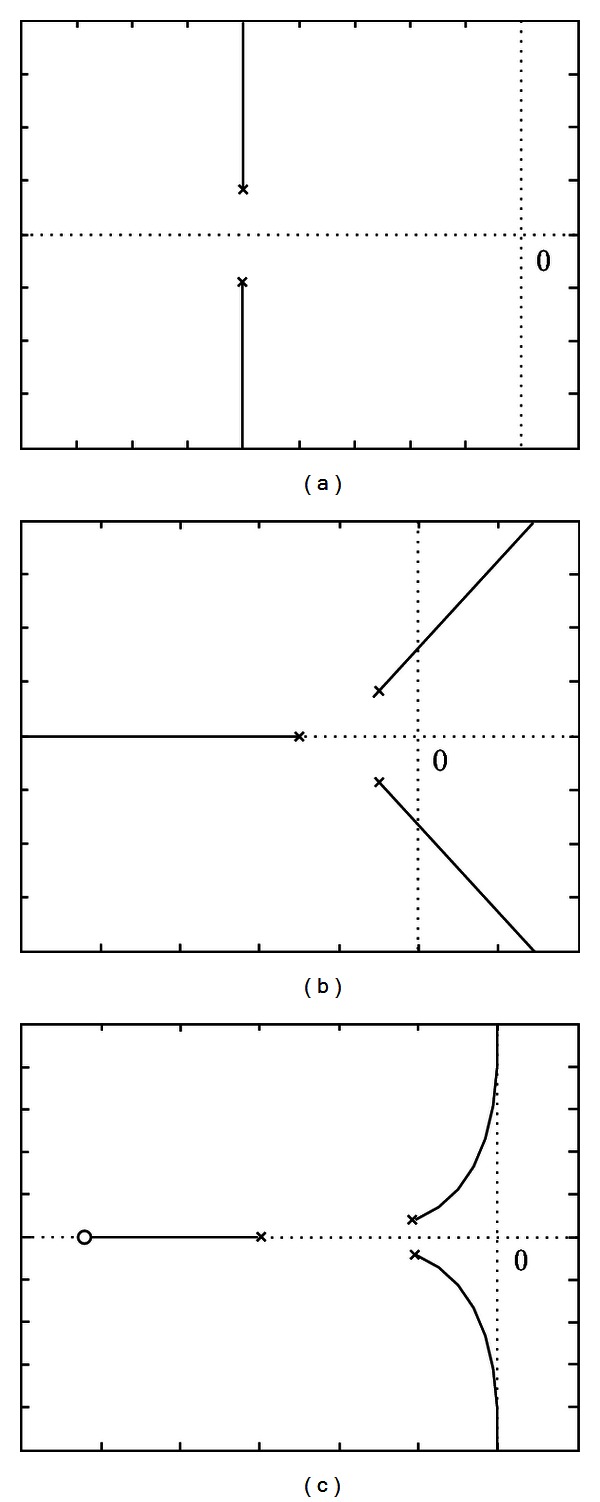
The root locus comparison before and after the compensation. (a) Origin root locus of the system. (b) Root locus of the system after step (1). (c) Root locus of the system after step (2).

If we put the open-loop transfer function (13) into a unit negative feedback system, the root locus of the closed-loop system is as Figure 2(a) shows. And the damping system is compensated on this basis. The basic idea is to make the unit step response of the compensated system become an equal amplitude oscillation through open-loop gain setting and pole-zero configuration. The problem is how to design the compensated system so that the root locus of the closed-loop system will pass through the imaginary axis, and at the same time, the operating point of the system is at the intersection of the root locus with the imaginary axis. A feasible solution to the problem is as follows.

1. Add a pole *s* = −*p* in the negative half real axis, where *p* > *ξω*_*n*_. After this step, the root locus of the system is as Figure 2(b) shows.
2. Considering that the compensated system is sensitive to the open-loop gain after step (1), then we add a zero *s* = −*z* to locate the asymptotes of the root locus in the right side of the imaginary axis. Let Δ*x* > 0 be the intersection of the asymptotes and the real axis. To meet the requirements of the asymptote (−*p* − 2*ξω*_*n*_ + *z*)/2 = Δ*x*, we get *z* = *p* +2*ξω*_*n*_ + 2Δ*x*, where Δ*x* should be moderately selected to avoid causing the open-loop gain to be too large. After this step, the root locus of the system is as Figure 2(c) shows.

According to the control theory, the closed-loop characteristic equation after the previous two steps is

(17) $$\begin{array}{c} \left(s+p\right)\left(s^{2}+2\xi \omega_{n}^{2}s+\omega_{n}^{2}\right)+K\omega_{n}^{2}\left(s+z\right)=0. \end{array}$$

The condition for a critical oscillation is that the poles of the closed-loop system are located on the imaginary axis. Letting *s* = *iy* and inserting it into (17), we have

(18) $$\begin{array}{c} K=\frac{\xi }{\omega_{n}\Delta x}\left(p^{2}+2\xi \omega_{n}p+\omega_{n}^{2}\right). \end{array}$$

Since *K*_0_ has been considered as a part in the open-loop gain *K*, that is, *K* = *K*′*K*_0_, we have

(19) $$\begin{array}{c} K^{\prime }=\frac{\xi }{\omega_{n}\Delta x}\left(p^{2}+2\xi \omega_{n}p+\omega_{n}^{2}\right)\left(\frac{4\gamma^{2}}{{\left|\Omega \right|}^{2}}+2\right). \end{array}$$

By inserting (12), (16) into (19), we get

(20) $$\begin{array}{c} K^{\prime }=\frac{3\gamma \left(p^{2}+3p\gamma +2\gamma^{2}+{\left|\Omega \right|}^{2}\right)}{\Delta x{\left|\Omega \right|}^{2}}. \end{array}$$

### 2.3. Physical Implementation

In the feedback control of the quantum system, the information of the density matrix cannot be measured directly because of the characteristics of the quantum system. One of the challenges exists in how to access the quantum information and feed it back to the input, in other words, how to construct the negative feedback channel for the quantum second-order system. In our work, for solving the problem of information acquisition of the quantum state, a quantum tomography scheme is designed to reconstruct the density matrix.

**Figure 3:**
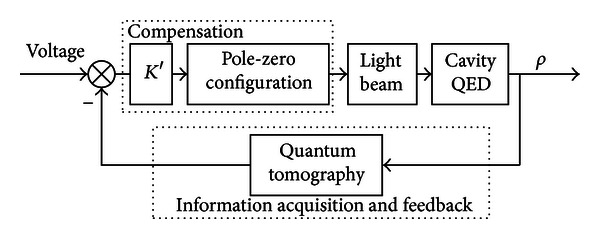
Schematic representation of the realization of the compensation network.

The physical implementation of the proposal is as Figure 3 shows. The detailed description of the steps is as follows: First, based on the tomography process, each element of the density matrix *ρ* is reconstructed from the output; thus, the quantum information has been transformed into classical information and fed back to the input. In order to realize the compensation strategy of the Rabi oscillation based on the transfer function analysis, a compensation circuit is designed using the active phase-lead compensator and the double integral A/D converter. The quantum density matrix information is transformed into the classical voltage signal for driving the light beam, which can be used to stabilize the Rabi oscillation. The details are described in Sections 2.3.1 and 2.3.2.

#### 2.3.1. Information Acquisition

Quantum tomography technique is an indirect method to determine quantum system parameters. The basic idea is to construct multiple copies of the photon from the system output and determine density matrix elements of the output photon through the optical operations of the photon copies [10]. Assuming that the system output is single-qubit photon, the measurement strategy is as follows [11, 12].

1. Let the *N* identical copies of the output photon pass through the horizontal-polarization wave plate, and record the number *n*_0_.
2. Let the *N* identical copies of the output photon pass through the vertically polarization wave plate and record the number *n*_1_.
3. Let the *N* identical copies of the output photon pass through the left-rotation wave plate and record the number *n*_2_.
4. Let the *N* identical copies of the output photon pass through the right-rotation wave plate and record the number *n*_3_.

The relationship between *n*_*i*_  (*i* = 0,1, 2,3) and the system output *ρ*_out_ can be described as follows [11, 12]:

(21) n0=N〈0|ρout|0〉,n1=N〈1|ρout|1〉,n2=N〈+|ρout|+〉,n3=N〈−|ρout|−〉.

The density matrix of output photon can be reconstructed according to (*n*_0_, *n*_1_, *n*_2_, *n*_3_):

(22) ρout=[n0n0+n1,n1n0+n1,(n2n0+n1−12)+i(n3n0+n1−12), (n2n0+n1−12)−i(n3n0+n1−12)]T.

#### 2.3.2. Compensation

If the element *ρ*_22_ of the density matrix has been reconstructed by (21), a voltage signal proportional to *ρ*_22_ can be obtained through the compensation to drive the light beam. The classical circuit to achieve this function consists of two parts.

**Figure 4:**
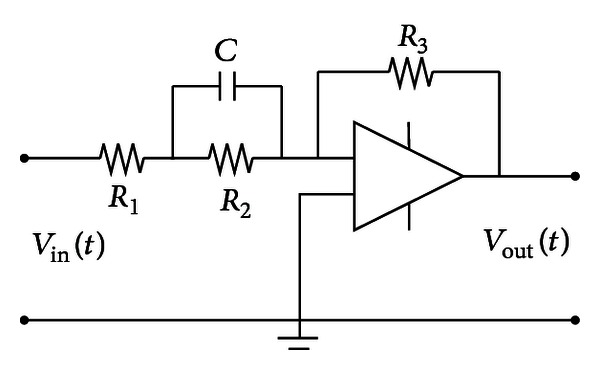
The active phase-lead compensation network.

**Figure 5:**
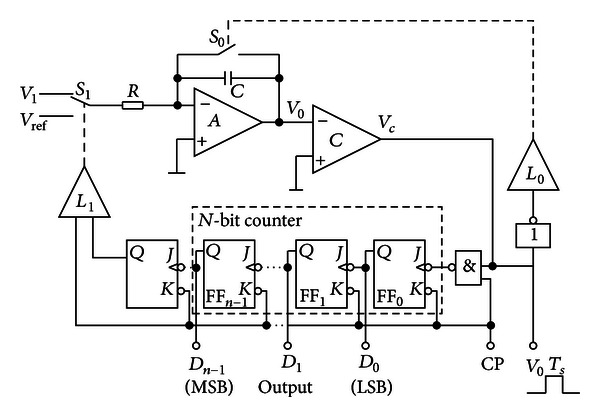
Double-integral A/D converters.

1. Due to the typical phase-lead compensation *D*(*s*) = (*s* + *z*)/(*s* + *p*), (*p* > *z*), the implementation of the active phase-lead compensation network is as Figure 4 shows.
2. Due to the 1/|*Ω*|^2^ in the expression of *K*′, a double integral A/D converter can be used to realize the reciprocal operation of |*Ω*|^2^ as Figure 5 shows.

## 3. Results and Discussion

Through the previous analysis, a coherence preserving solution in cavity QED has been presented using the quantum tomography and the Rabi oscillation compensation. In the following, simulation results have been analyzed for the evaluation of the strategy.

### 3.1. Results of the Quantum Tomography

As stated in Section 2.3, we will let the copied output photons pass through four types of polarization wave plate and record the number of the photons passing through each type of the wave plate, respectively, for reconstructing the output density matrix. Taking into account the errors that may exist in the process, we should mention that there are mainly two kinds of errors in any realistic system: the first is the measurement error due to the accuracy and sensitivity of the experimental apparatus, the noise from the external environment, the random interference, and so forth; and the other one is the statistical error caused by the random collapse because of the measurement of the output state; that is, infinite times of detection are needed to obtain the accurate quantum information theoretically, which is impossible in practice. 

In this simulation, a theoretical value of *n*_*i*_  (*i* = 0,1, 2,3) can be calculated from (21). In order to investigate the error's effect on the reconstruction, a random interference is added on each value of *n*_*i*_  (*i* = 0,1, 2,3) for calculating the output density matrix by (22). We can get the difference of each component of the density matrix between the target quantum state *ρ*_*ij*_ and the reconstructed quantum state *ρ*_*ij*_′ by

(23) $$\begin{array}{c} \Delta \rho_{ij}=\left|\rho_{ij}-\rho_{ij}^{\prime }\right|, \end{array}$$

where the operator |·| stands for the magnitude of the error and as for the single-qubit quantum state, *i*, *j* = 1,2.

**Figure 6:**
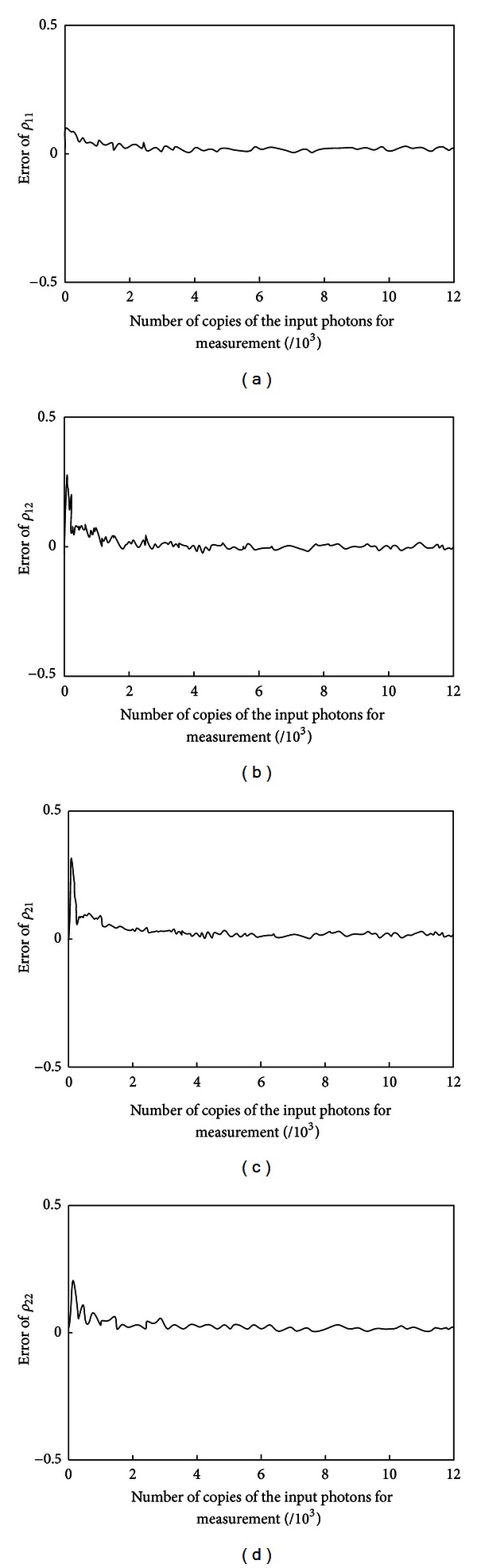
The error of each component of the reconstructed density matrix. (a) The error of *ρ*_11_. (b) The error of *ρ*_12_. (c) The error of *ρ*_21_. (d) The error of *ρ*_22_.

In this paper, a series of error data are obtained by changing the number of the input photons. The relationship between the error and the number of the input photon is shown in Figure 6, from which the following conclusions can be drawn.

1. Due to the presence of measurement error in the experiment, there is deviation between the statistical number of photons and the theoretical value, which will lead to the deviation between the reconstructed density matrix and the target quantum state density matrix. Therefore, the reconstructed density matrix may not satisfy the conditions of completely positive or preserving the trace, and this deviation is randomly generated and will have an inevitable impact on the results, which is more significant especially when the number of input photons is relatively small.
2. According to the theory of quantum tomography, infinite times of detection are needed to guarantee a completely accurate reconstructed density matrix, which cannot be done in the experiment. However, as can be seen from the simulation results in Figure 6, with the number of photons increasing, the error would converge to a satisfactory extent; in particular, when the number of input photons reaches 3,000 or more, it is reasonable to say that the negative impact of the measurement errors and statistical errors is negligible.
3. Though the error can be eliminated by infinitely increasing the number of input photons, doing so will significantly increase the cost and reduce the efficiency of the experiment. An appropriate number of input photons should be chosen to achieve a compromise between the error and efficiency.

### 3.2. Results of the Rabi Oscillation Stabilization

**Figure 7:**
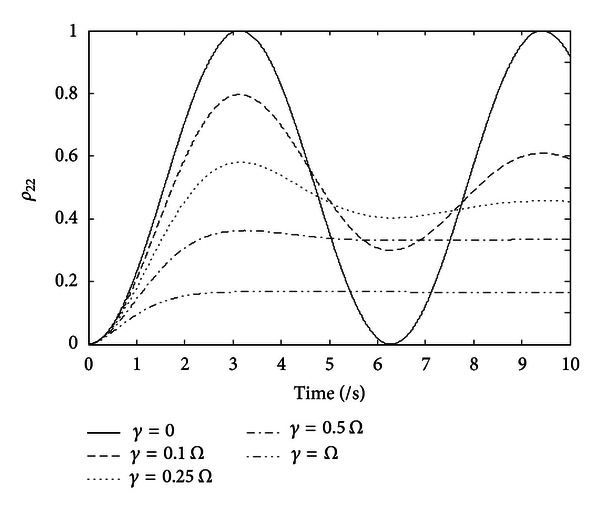
Rabi oscillation with spontaneous emission.

From the discussion in Section 2, in the atom light interactions, if there is no damping due to the spontaneous emission, the process is coherent and reversible. But if the decoherence caused by the spontaneous emission exists, in other words, *γ* ≠ 0, the equal amplitude oscillation will change to a damped one, which will generate detrimental effects on quantum information processing. The impact of the decoherence caused by the spontaneous emission is as Figure 7 shows. At *t* = 0, the atom is in the ground state (*ρ*_11_(0) = 1,  *ρ*_22_(0) = 0). The probability to find the atom in the excited state is plotted for various ratios of *γ* and the Rabi frequency *Ω*. In the simulation, we have chosen the following value: *γ* = 0,  *γ* = 0.1*Ω*,  *γ* = 0.25*Ω*,  *γ* = 0.5*Ω*, and *γ* = *Ω*.

As can be seen from Figure 7, when *γ* = 0, the oscillation is with equal amplitude and fixed frequency. With the *γ* increasing, the damping effect becomes more and more significant. The main objective of our design is to overcome the damping effect by compensation; that is to say, when *γ* which stands for the spontaneous emission exists, the process will still be a coherent process with equal amplitude and fixed frequency.

**Figure 8:**
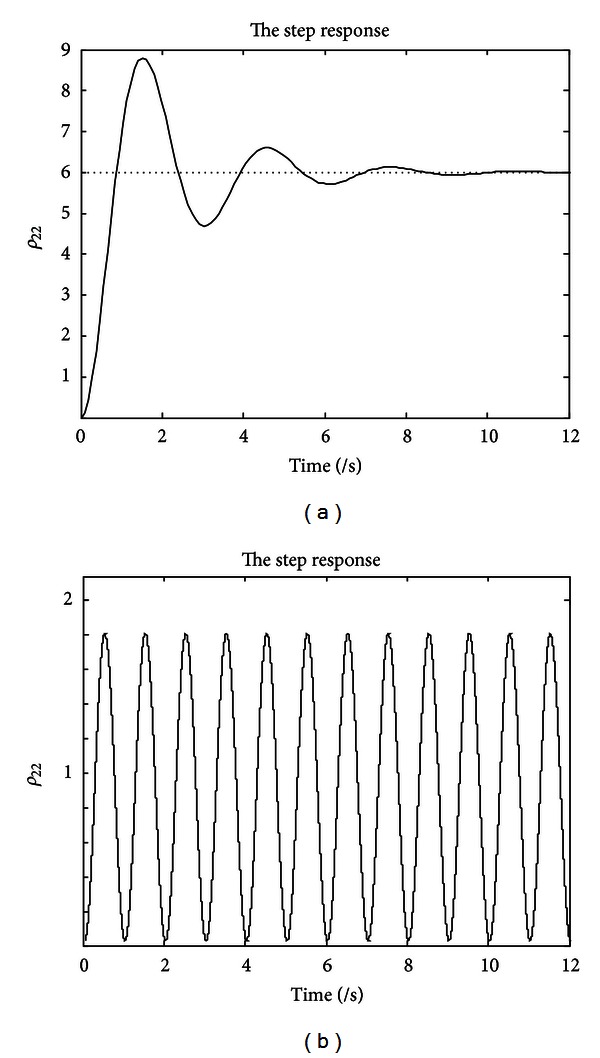
The step response comparison before and after the compensation. (a) The step response of the original system. (b) The step response after the compensation.

According to the strategy described in Section 2, taking *γ* = 0.3, |*Ω* | = 2, *p* = 1.5, and Δ*x* = 0.1, the unit step response of the uncompensated and compensated system is shown in Figures 8(a) and 8(b), respectively.

It is clear that the uncompensated system approaches a constant value after the damping process and the compensated system can maintain a sustained oscillation with a constant frequency and amplitude, which have proved the effectiveness and superiority of our design.

## 4. Conclusions

For the coherence preservation in cavity QED, the model of the damping Rabi oscillation in the form of the transfer function is derived based on the optical Bloch equation. The transfer function of the damping Rabi oscillation is compensated using the root locus technique derived from the classical control theory to suppress the atom's spontaneous emission. Finally, a physical implementation is put forward to keep the coherence in cavity QED. The strategy has provided a basis for the entanglement preparation in cavity QED theoretically. The research has theoretical significance and practical value. The simulation results showed that the compensated system can maintain a sustained oscillation with a constant frequency and amplitude. And it means the process is a coherent reversible one, which is an ideal environment for quantum information processing.

However, this work is based on the semiclassical optical Bloch equations. In other words, only the atom is quantized and the field is treated as a definite function of time rather than as an operator. To obtain more rigorous results, the further work will focus on the Jaynes-Cummings model, in which the radiation field is also quantized. And meanwhile, an appropriate quantum feedback channel [13] is expected to be found to replace the current classical feedback channel.
